# Evaluation of consciousness rehabilitation via neuroimaging methods

**DOI:** 10.3389/fnhum.2023.1233499

**Published:** 2023-09-14

**Authors:** Jianyang Wang, Xinyu Gao, Zuchao Xiang, Fangfang Sun, Yong Yang

**Affiliations:** College of Automation, Hangzhou Dianzi University, Hangzhou, China

**Keywords:** disorders of consciousness, EEG, fMRI, PET, fNIRS, multimodal

## Abstract

Accurate evaluation of patients with disorders of consciousness (DoC) is crucial for personalized treatment. However, misdiagnosis remains a serious issue. Neuroimaging methods could observe the conscious activity in patients who have no evidence of consciousness in behavior, and provide objective and quantitative indexes to assist doctors in their diagnosis. In the review, we discussed the current research based on the evaluation of consciousness rehabilitation after DoC using EEG, fMRI, PET, and fNIRS, as well as the advantages and limitations of each method. Nowadays single-modal neuroimaging can no longer meet the researchers` demand. Considering both spatial and temporal resolution, recent studies have attempted to focus on the multi-modal method which can enhance the capability of neuroimaging methods in the evaluation of DoC. As neuroimaging devices become wireless, integrated, and portable, multi-modal neuroimaging methods will drive new advancements in brain science research.

## 1. Introduction

Disorders of consciousness (DoC) generally refers to changes in arousal or awareness caused by severe brain injury (Zhu et al., 2019). DoC mainly includes coma, unresponsive wakefulness syndrome/vegetative state (UWS/*VS*) (Laureys et al., 2010), and minimally conscious state (MCS). Coma refers to a state characterized by the complete absence of arousal or awareness. UWS/*VS* is defined as arousal without awareness and MCS is defined as minimal, reproducible but inconsistent awareness (Edlow et al., 2021). MCS can be further classified into MCS+ and MCS-, depending on the absence or presence of high-level behavioral responses such as command-following (Aubinet et al., 2020). The consciousness state of most DoC patients is difficult to alter and will remain in a particular state for months or years, which places a considerable burden on families and society. Accurate evaluation of consciousness rehabilitation is crucial for personalized and precise treatment on DoC patients.

Behavioral assessment scales are widely used in clinical settings and the coma recovery scale revised (CRS-R) has become the gold standard for evaluation the consciousness rehabilitation of DoC patients (Giacino et al., 2004; Arico et al., 2016). The CRS-R includes 29 items which are categorized into six subscales that assess auditory, visual, motor, oro-motor, communication, and arousal processes. A score of ≤2 on the auditory, motor, and oro-motor/verbal subscales, ≤1 on the visual subscale, and 0 on the communication subscale is indicative of a diagnosis of UWS/VS. Higher scores suggest a condition of MCS, whereas reaching a score of 6 in the motor subscale and 2 in the communication subscale signifies a transition out of the MCS state.

**Figure 1:**
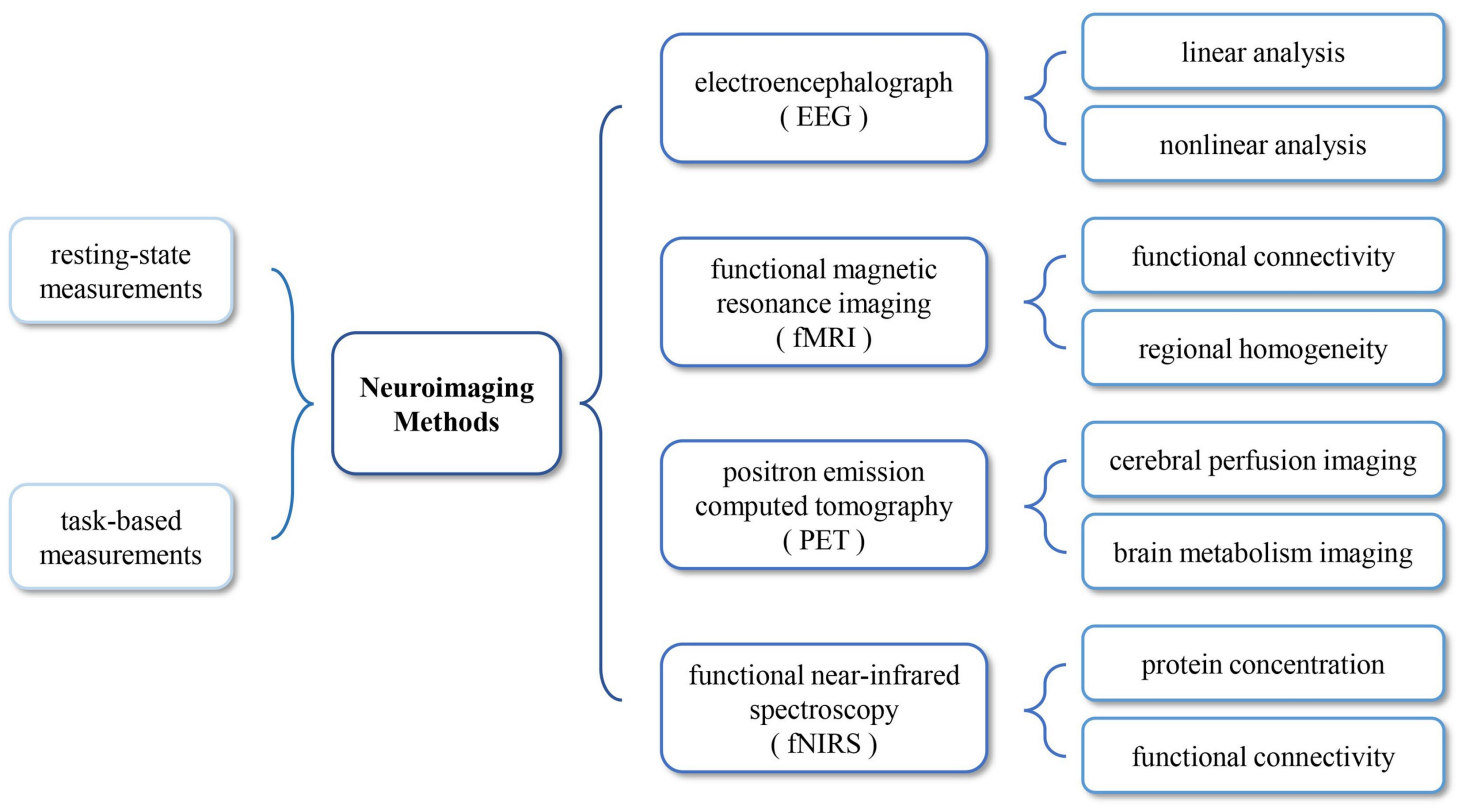
Various neuroimaging methods.

However, misdiagnosis remains a serious issue, a study (Kondziella et al., 2016) shows that approximately 40% of DoC patients are misdiagnosed as UWS due to their inability to communicate, since patients’ level of consciousness could fluctuate due to the passage of time and influence of external stimuli, and the scoring of the scale is susceptible to subjective factors. An increasing number of studies indicated that neuroimaging methods can show residual brain activity in patients who have no evidence of consciousness in behavioral or clinical assessments. These patients consciously regulate their brain activity in response to commands and even answer “yes” or “no” questions by performing mental imagery tasks (Scolding et al., 2021; Thibaut et al., 2021). Neuroimaging methods for consciousness rehabilitation evaluation is divided into two categories: resting-state and task-based paradigms (Figure 1). The resting-state paradigms detect spontaneous brain activity in patients without any stimulation, while the task-based paradigms detect brain activity changes in response to external stimuli or by having patients perform specific tasks. Neuroimaging methods do not rely on subjective judgment and assist doctors with objective, quantifiable characteristics to evaluate patients’ consciousness rehabilitation. Hopefully, neuroimaging methods will improve the survival rate of DoC patients.

**Figure 2:**
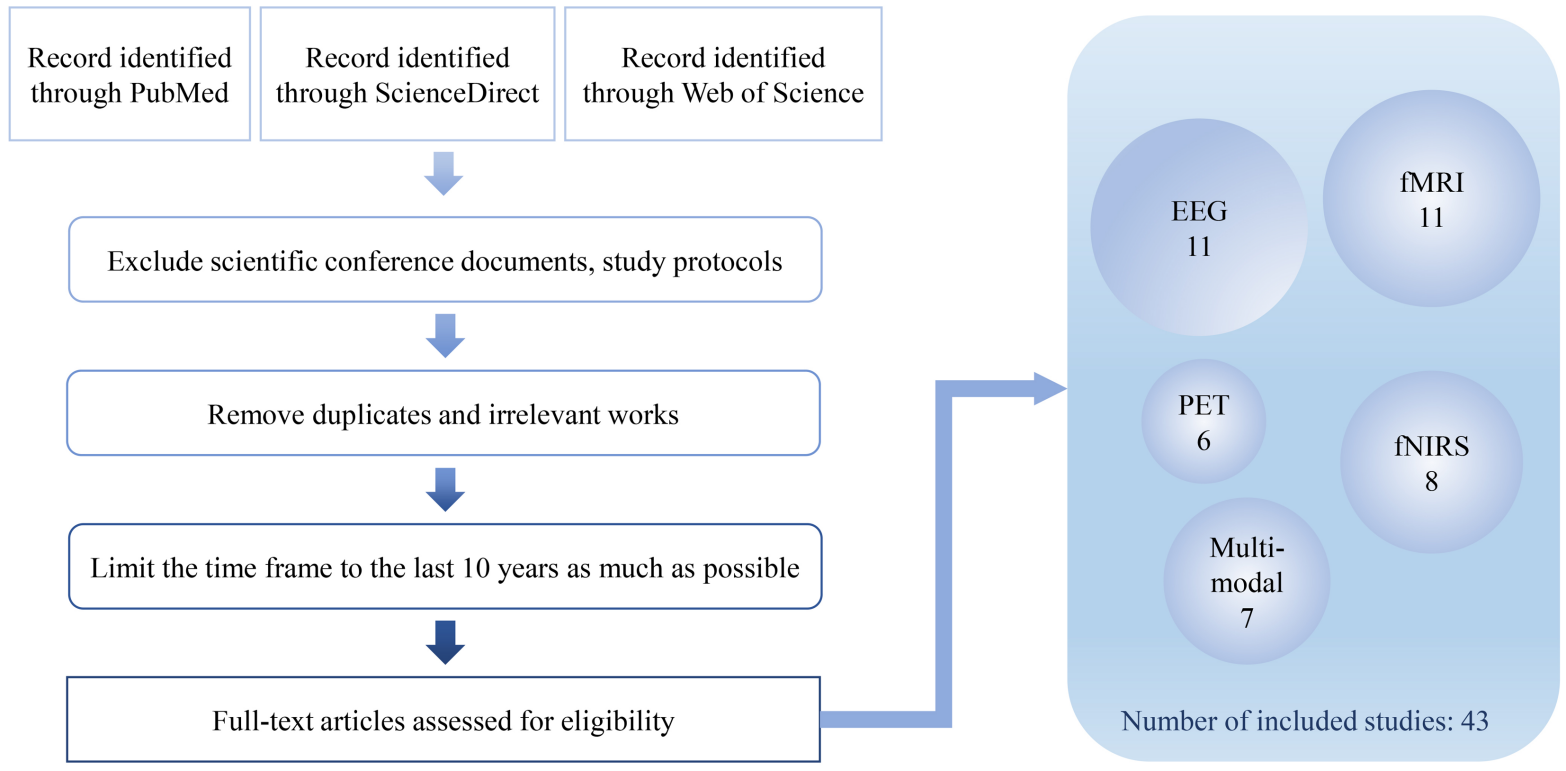
Flow chart of study selection process.

The paper reviews the application of neuroimaging methods in the evaluation of consciousness rehabilitation of DoC patients, including traditional electroencephalography (EEG), functional magnetic resonance imaging (fMRI), positron emission computed tomography (PET), and burgeoning functional near-infrared spectroscopy (fNIRS). A search in PubMed, ScienceDirect and Web of Science was conducted using search terms (disorders of consciousness OR vegetative state OR unresponsive wakefulness syndrome OR minimally conscious state) AND (neuroimaging OR EEG OR fMRI OR PET OR fNIRS OR multi-modal). Scientific conference documents, study protocols are excluded. After removing duplicates and irrelevant works, the search results were limited to the last 10 years as far as possible. The final search results in 43 relevant works that were available for full-text download (Figure 2). More information about these studies is summarized in Table 1. The advantages and limitations of each method are discussed and the potential research directions are provided. Due to the inherent limitations of single techniques, an increasing number of studies are adopting multi-modal methods to evaluate consciousness rehabilitation in DoC patients. The review paper also discusses the potential and challenges faced by multi-modal neuroimaging methods, which offers new perspectives for the clinical assessment of DoC patients using neuroimaging methods.

**Table 1:** Summary of included studies.

Author (Year)	Sample size	Method	Improvement criteria	Paradigms	Context	Findings
Schorr et al. (2016)	73 MCS/UWS and 24 HC	EEG	CRS-R beginning/after 12 moths	Resting-state	Investigated differences of EEG coherence within (short-range), and between (long-range) specified brain areas as well as EEG power over frontal, parietal, occipital, and temporal areas.	Fronto-parietal and parietal coherence could positively predict improvement of UWS patients
Lehembre et al. (2012)	21 MCS and 10 *VS*/UWS	EEG	CRS-R	Resting-state	Power spectra were obtained and three connectivity measures (coherence, the imaginary part of coherency and the phase lag index) were computed.	Connectivity measures were correlated with the CRS-R diagnosis; patients in the *VS*/UWS had significantly lower connectivity than MCS patients in the theta and alpha bands.
Naro et al. (2018)	17 UWS and 15 MCS	EEG	CRS-R	Resting-state	Assessed dynamic functional connectivity (DFC) in the resting state by analyzing the time-dependent EEG phase synchronization in five frequency bands.	MCS Patients showed changes in DFC matrices and topography over time, which were significantly different from those observed in UWS patients. The degree of DFC significantly correlated with the level of behavioral responsiveness measured using CRS-R.
Stefan et al. (2018)	42 *VS*/MCS	EEG	CRS-R	Resting-state	Applied microstates, entropy, power in alpha and delta frequency bands, and connectivity (weighted symbolic mutual information, symbolic transfer entropy, complex network analysis) to resting-state EEG data.	Several mathematically precise biomarkers perform significantly better than expected by chance at predicting outcome of coma, with the most promising results obtained through the analysis of EEG signals represented as microstates.
Kempny et al. (2018)	5 *VS*/UWS, 11 MCS and 12 HC	EEG/ERP	GCS RCP SMART	Task-based	Used as the ERP stimuli the subjects’ own name, others’ names and reversed other names and performed a sensor level analysis using SPM	This study shows the feasibility of using self-relevant stimuli such as a subject’s own name for assessment of brain function in pDOC patients.
Wu et al. (2018)	7 UMS, 7 MCS and 14 HC	EEG	CRS-R GOS	Task-based	Brain response to music, subjects’ own name, and noise was monitored and predictive QEEG values in various brain regions were investigated.	A subject’s own name might be an effective awakening therapy for patients with disorders of consciousness. Moreover, QEEG index in specific stimulation states may be used as a prognostic indicator for DoC patients.
Li et al. (2018)	10 *VS* and 9 MCS	EEG	CRS-R GOS	Task-based	Wavelet transformation and nonlinear dynamics were used to extract the features of EEG signals and four brain lobes were selected to investigate the degree of EEG response to habit stimulation.	Results showed that the highest degree of EEG response was from the call-name stimulation, followed by habit and music stimulations. These findings prove that habit stimulation induces relatively more intense EEG responses in DoC patients than music stimulation.
Sinitsyn et al. (2020)	11 UWS and 13 MCS	EEG	CRS-R	Task-based	Tested the reliability of PCI in an independently collected sample of 24 severely brain-injured patients.	The individual maximum PCI value across stimulation sites fell within the consciousness range in 11 MCS patients, yielding a sensitivity of 92% that surpassed qualitative evaluation of resting EEG. Most UWS patients showed a slow and stereotypical TMS-EEG response, associated with low-complexity PCI values.
Zhang et al. (2022)	18 UWS, 29 MCS and 19 HC	EEG	CRS-R	Task-based	ERP, scalp topography, and time-frequency maps were analyzed. The coherence and debiased weighted phase lag index networks in the delta, theta, alpha, beta, and gamma bands were constructed, and the correlations of network properties and CRS-R motor function scores were calculated.	The node degree properties of the COH network in the theta and alpha bands and the global efficiency properties of the dwPLI network in the theta band were significantly greater in the MCS group compared to the UWS group. The network properties and CRS-R motor function scores showed a strong linear correlation.
Di Gregorio et al. (2022)	15 TBI and 18 Non-TBI	EEG	CRS-R GOS GOSE	Resting-state	Extracted a set of EEG biomarkers in 33 DoC patients with traumatic and nontraumatic etiologies and estimated their accuracy to discriminate patients’ etiologies and predict clinical outcomes 6 months after the injury.	Machine learning reached an accuracy of 83.3% with EEG-based functional connectivity predicting clinical outcome in nontraumatic patients. Furthermore, the combination of functional connectivity and dominant frequency in EEG activity best predicted clinical outcomes in traumatic patients with an accuracy of 80%.
Wang et al. (2022)	105 UWS, 76 MCS and 30 HC	TMS-EEG	CRS-R beginning/after 1 year	Resting-state	Applied fast perturbational complexity index (PCIst) to the diagnosis and prognosis of DOC patients and trained the classification and regression model.	PCIst shows the differences among NOR, MCS and UWS and has low computational cost. PCIst at 9–12 Hz shows the highest performance in diagnosis and prognosis of DOC.
Di et al. (2007)	7 *VS* and 4 MCS	fMRI	CRS-R at the time of scanning, and 1, 2, and 3 months after fMRI acquisition	Resting-state	To evaluate the differences in brain activation in response to presentation of the patient’s own name spoken by a familiar voice (SON-FV) in patients with *VS* and MCS. prospectively studied residual cerebral activation to SON-FV by using fMRI in seven patients with *VS* and four with MCS.	The cerebral responses to patient’s own name spoken by a familiar voice as measured by fMRI might be a useful tool to preclinically distinguish MCS.
Fernandez-Espejo et al. (2008)	3 *VS*, 4 MCS and 19 HC	fMRI	GCS DRS LCFS	Task-based	Analyzed cerebral response to language and to complex sound processing in the healthy subjects’ group and in each patient, using SPM5.	Some patients in *VS* and MCS can preserve cerebral responses to language and auditory stimuli.
Okumura et al. (2014)	5 *VS*, 2 MCS and 21 HC	fMRI	CRS	Task-based	Examined the brain regions activated by music and assessed topographical differences of the music stimulation-activated brain among healthy adults and patients.	The presence of STG activation by MS may predict a possible improvement of patients in a *VS* to MCS.
Wang et al. (2015)	39 *VS*, 25 MCS and 2 EMCS	fMRI	CRS-R	Task-based	the response of each subject to his/her own name said by a familiar voice (SON-FV) was recorded using fMRI;	The activation type and volume in auditory cortex elicited by SON-FV significantly correlated with *VS*/UWS patients’prognosis, particularly in patients with traumatic etiology.
Boltzmann et al. (2021)	13 UWS and 18 HC	fMRI	CRS-R	Task-based	Both groups were exposed to different auditory stimulation (scanner noise, preferred music, and aversive auditory stimulation). Functional connectivity was analyzed using a seed-based approach	UWS patients showed impaired functional connectivity within all resting-state networks. In addition, functional connectivity of the auditory network was modulated by preferred music and aversive auditory stimulation.
Liang et al. (2014)	5 UWS/MCS and 11 HC	fMRI	GCS GOS	Task-based	Speech comprehension, mental imagery, and question–answer tests were performed.	The results from 2 patients provide independent support of similar work by others with 3 T fMRI, and demonstrate broader clinical utility for these tests at 1.5 T despite lower signal-to-noise ratio.
Wang et al. (2019)	8 MCS, 21 *VS*/UWS and 15 HC	fMRI	CRS-R	Task-based	Analyzed the command-following responses for robust evidence of statistically reliable markers of motor execution.	Activity of the motor-related network may be taken as an indicator of high-level cognition that cannot be discerned through conventional behavioral assessment.
Bodien et al. (2017)	4 UWS, 5 MCS, 1 Coma and 10 HC	fMRI	CRS-R GOS	Task-based	Compared the rate of covert consciousness detection by hand squeezing and tennis playing motor imagery paradigms.	The tennis paradigm performed better than the hand squeezing paradigm in healthy subjects, but in patients, the hand squeezing paradigm detected command following with greater ACC.
Luppi et al. (2019)	22 UWS/MCS and 16 HC	fMRI	CRS-R	Resting-state	Combined graph theory and dynamic functional connectivity to compare resting-state functional MRI data from awake volunteers, propofol-anaesthetised volunteers, and patients with disorders of consciousness.	Cortical networks are especially affected by loss of consciousness during temporal states of high integration, exhibiting reduced functional diversity and compromised informational capacity, whereas thalamo-cortical functional disconnections emerge during states of higher segregation. Spatially, posterior regions of the brain’s default mode network exhibit reductions in both functional diversity and integration with the rest of the brain during unconsciousness.
Vanhaudenhuyse et al. (2010)	4 UWS, 4 MCS, 1 LIS, 5 Coma and 14 HC	fMRI	CRS-R GLS	Resting-state	Connectivity was investigated using probabilistic independent component analysis, and an automated template-matching component selection approach.	Connectivity in all default network areas was found to be negatively correlated with the degree of clinical consciousness impairment, ranging from healthy controls and locked-in syndrome to minimally conscious, vegetative then coma patients.
Huang et al. (2020)	23 + 14 + 12 + 13 *VS*/UWS, 8 MCS and 28 HC	fMRI	CRS-R	Resting-state	Examined how the default mode network and the dorsal attention network are regulated in the conscious brain and how they are disrupted when consciousness is diminished.	Provided evidence for a “temporal circuit” characterized by a set of trajectories along which dynamic brain activity occurs. We demonstrate that the transitions between default mode and dorsal attention networks are embedded in this temporal circuit, in which a balanced reciprocal accessibility of brain states is characteristic of consciousness.
Qin et al. (2021)	12 + 24 + 17 + 6 + 50 UWS/MCS/BI	EEG fMRI	CRS-R	Resting-state	Used a graph-theoretical measure of degree centrality conjoined with ROI-based functional connectivity.	Regions forming a higher-order sensorimotor integration circuit are involved in supporting consciousness within the brain’s global functional network.
Laureys et al. (2002)	15 PVS and 15 HC	PET	GCS	Task-based	Changes in regional cerebral blood flow were measured during high-intensity electrical stimulation of the median nerve compared with rest in 15 nonsedated patients and in 15 healthy controls. Evoked potentials were recorded simultaneously.	Somatosensory stimulation of PVS patients, at intensities that elicited pain in controls, resulted in increased neuronal activity in primary somatosensory cortex, even if resting brain metabolism was severely impaired. However, this activation of primary cortex seems to be isolated and dissociated from higher-order associative cortices.
Bruno et al. (2010)	10 *VS*(5 patients without fixation and 5 presented visual fixation) and 39 HC	PET	CRS-R	Task-based	FDG-PET data were pre-processed and analyzed using Statistical Parametric Mapping. A psychophysiological interaction analysis was used to test for differences in functional cortico-cortical connectivity in patients without and with visual fixation.	Sustained visual fixation in (non-traumatic) disorders of consciousness does not necessarily reflect consciousness and higher order cortical brain function.
Schiff et al. (2002)	5 PVS	PET MRI MEG	/	Resting-state	Employed [18F]fluorodeoxyglucose-positron emission tomography (FDG-PET), MRI and magnetoencephalographic (MEG) responses to sensory stimulation to study five PVS patients with different behavioural features,.	The specific patterns of preserved metabolic activity identified in these patients do not appear to represent random survivals of a few neuronal islands; rather they reflect novel evidence of the modular nature of individual functional networks that underlie conscious brain function.
Rudolf et al. (2002)	9 AVS and 9 HC	PET	AAN	Resting-state	Compared the results of parallel PET studies of regional cerebral glucose metabolism with the radiotracer 18F- FDG and BZR density by PET using the BZR	Alterations of cerebral glucose consumption do not represent mere functional inactivation, but irreversible structural brain damage.
Usami et al. (2022)	15 *VS*, 15 MCS- and 20 MCS+	PET	JFK	Resting-state	Measured FDG-PET-based CMRGlu values in 12 regions of both brain hemispheres and compared those among MCS+, MCS- and *VS* patients.	Functional preservation in the left occipital region in patients with chronic DOCs might reflect an awareness of external environments, whereas extensive functional preservation in the right cerebral hemisphere might reflect communication motivation.
Yamaki et al. (2023)	2 UWS, 9 MCS-, 3 MCS+ and 3 patients with severe disability	PET	GCS	Resting-state	Investigated whether regional brain glucose metabolism assessed by 18F- FDG-PET at rest could predict voluntary movement in severe TBI patients,.	FDG-PET may be a useful and robust biomarker for predicting long-term recovery of motor function in severe TBI patients with disorders of consciousness.
Hattori et al. (2003)	23 TBI and 11 HC	PET	GCS	Resting-state	The CMRglc of cortical areas (remote from hemorrhagic lesions), striatum, thalamus, brain stem, cerebellar cortex, and whole brain was compared with severity of injury and the level of consciousness evaluated using GCSini and the Glasgow Coma Scale score at the time of PET (GCSpet).	Demonstrated a significant difference in glucose metabolism in the thalamus, brain stem, and cerebellum between comatose and noncomatose patients acutely after TBI. The metabolic rate of glucose in these regions significantly correlated with the level of consciousness at the time of PET.
Hermann et al. (2021)	21 *VS*, 31 MCS and 32 HC	PET EEG	CRS-R	Resting-state	Compared out-sample diagnostic and prognostic performances of PET-MIBH and EEG-based classification of conscious state to the current behavioral gold-standard CRS-R.	FDG-PET MIBH is an accurate and robust procedure across sites to diagnose MCS. Its combination with EEG-based classification of conscious state not only optimizes diagnostic performances but also allows to detect covert cognition and to predict 6-month command-following recovery.
Luppi et al. (2022)	21 DOC patients and 20 HC	PET MRI	/	Resting-state	Developed and systematically perturbed a neurobiologically realistic model of whole-brain haemodynamic signals.	By incorporating PET data about the cortical distribution of GABA receptors, the computational model reveals a key role of spatially-specific local inhibition for reproducing the functional MRI activity observed during anaesthesia with the GABA-ergic agent propofol.
Liu et al. (2023a)	11 UWS and 12 MCS	fNIRS	CRS-R	Resting-state	Graph-theoretical analysis and seed-based correlation analyses were used to investigate the network topology and the strength of pairwise connections between ROIs and channels.	MCS and UWS have different patterns of topological architecture and short- and long-distance connectivity in PFC. Intraconnections within BA10 and interhemispheric connections between BA10 and 46 are excellent resting-state fNIRS classifiers for distinguishing between MCS and UWS.
Shu et al. (2023)	10 DoC patients	fNIRS	CRS-R	Resting-state	Functional connectivity analysis quantified the communication between brain regions, area communication strength, and global communication efficiency. Linear regression analysis was conducted between the changes of indices based on functional connectivity analysis and the changes of the CRS-R index.	Patients with trauma exhibited a greater increase of CRS-R scores after DBS treatment compared with patients with hemorrhage and brainstem infarction. Global communication efficiency changed consistently with the CRS-R index.
Kempny et al. (2016)	5 *VS*/UWS, 11 MCS and 10 HC	fNIRS	GCS	Task-based	Evaluated the brain function of patients with pDOC by using a motor imagery task while recording NIRS.	This study demonstrated the feasibility of using NIRS for the assessment of brain function in pDOC patients using a motor imagery task.
Li et al. (2021)	5 MCS and 6 HC	fNIRS	CRS-R	Task-based	An active command-driven motor imagery (MI) paradigm based on fNIRS was used to detect residual consciousness in patients with prolonged DOC.A support vector machine (SVM) classifier was used to classify yes-or-no responses.	This study confirmed the feasibility of using portable fNIRS technology to detect residual cognitive ability in patients with prolonged DOC by active command-driven motor imagery.
Kurz et al. (2018)	2 MCS and 10 HC	fNIRS	CRS-R	Task-based	Evaluated the applicability of auditory presented mental-arithmetic tasks using fNIRS.	Despite inconsistent correlations between the patient data and the HC group, single runs of the patient recordings revealed task-synchronous patterns.
Si et al. (2018)	10 DoC patients	fNIRS	JFK	Task-based	In this pilot study, fNIRS was used to measure the hemodynamic responses of 10 DOC patients to different SCS frequencies (5 Hz, 10 Hz, 50 Hz, 70 Hz, and 100 Hz).	SCS modulates the hemodynamic responses and long-range connectivity in a frequency-specific manner (with 70 Hz apparently better), perhaps by improving the cerebral blood volume and information transmission through the reticular formation-thalamus-cortex pathway.
Zhang et al. (2018)	7 *VS* and 2 MCS	fNIRS	JFK GOS	Task-based	Monitored the blood volume fluctuations in the prefrontal and occipital cortices during the SCS using fNIRS,.	A shorter ISI was found to improve the blood volume in the prefrontal cortex and this phenomenon was more significant for the subgroup of patients with a favorable prognosis.
Bicciato et al. (2022)	6 HC	fNIRS	/	Task-based	Investigated variations in the oscillatory signal of fNIRS in the spectral regions of low-frequency (LFO) and very-low-frequency oscillations (VLFO).	The first fNIRS study on the cortical hemodynamic response to favorite music using a frequency domain approach. The results could identify a typical pattern of brain response to music.
Cavaliere et al. (2018)	1 UWS, 1 MCS and 1 EMCS	PET fMRI	CRS-R	Resting-state	Presented findings obtained by hybrid fludeoxyglucose (FDG-)PET/MR imaging in three severely brain-injured patients.	Combined PET/fMRI analysis demonstrated a higher functional/metabolic correlation for patients in EMCS and MCS compared to UWS.
Amiri et al. (2023)	51 UWS and 36 MCS	EEG fMRI	GCS	Resting-state	Used EEG and fMRI data at study enrolment in two different machine-learning algorithms (Random Forest and SVM with a linear kernel) to distinguish patients in a minimally conscious state or better (≥MCS) from those in coma or unresponsive wakefulness state (≤UWS) at time of study enrolment and at ICU discharge (or before death).	Suggested that acute DoC prediction models in the ICU be based on a combination of fMRI and EEG features, regardless of the machine-learning algorithm used.
Othman et al. (2021)	9 patients and 14 controls	EEG NIRS	FOUR	Resting-state	Explored resting-state oscillations in eight-channel NIRS oxyhemoglobin and eight-channel EEG band-power signals to assess neurovascular coupling.	Neurovascular coupling between NIRS oxyhemoglobin (0.07–0.13 Hz) and EEG band-power (1–12 Hz) signals at frontal areas was sensitive and prognostic to changing consciousness levels.

## 2. EEG used in DoC studies

**Figure 3:**
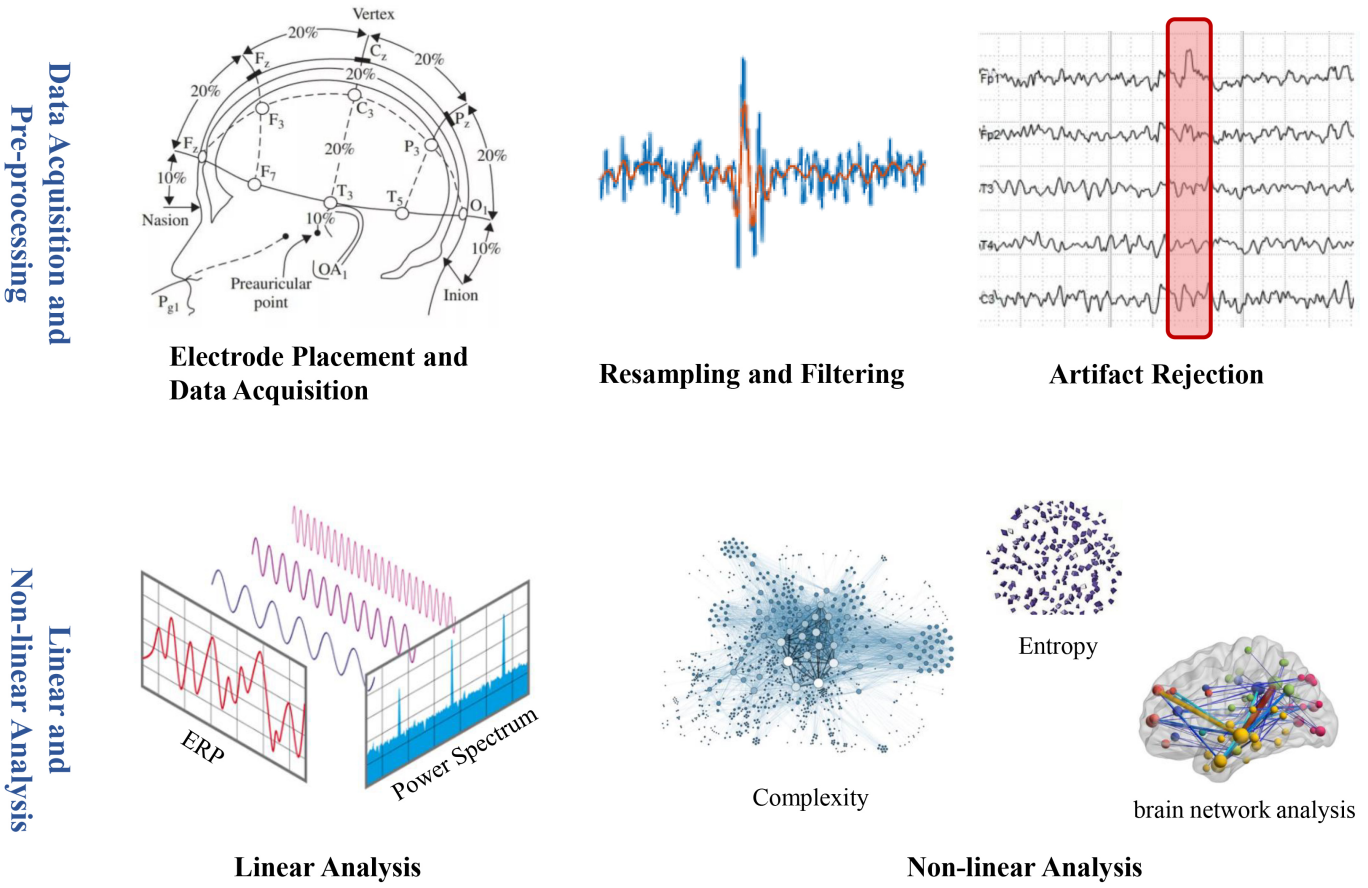
EEG acquisition and analysis.

With the advantage of being economical and portable, EEG has been applied earlier in the evaluation of consciousness rehabilitation among various neuroimaging methods (Ballanti et al., 2022). The brain’s neurons communicate with each other through electrical impulses, which generate small electrical currents. These electrical currents can be detected on the scalp as voltage fluctuations using the EEG electrodes. EEG studies in DoC patients can be mainly divided into two main methods: linear and nonlinear analysis (Wu et al., 2020). The linear analysis reflects brain function by extracting information in the time or frequency domain of EEG signals, such as event-related potential (ERP), power spectral density (PSD), coherence, etc. Nonlinear dynamics features can represent the intensity of neuronal activity in brain waves and brain regions, which allows the brain to be viewed as a very complex chaotic system (Theiler et al., 1992). The nonlinear analysis uses analytical methods such as sample entropy (SampEn), fuzzy entropy (FE), permutation entropy (PE), Lempel-Ziv complexity (LZC), detrended fluctuation analysis (DFA), etc (Figure 3).

### 2.1. Task-based EEG paradigms in DoC studies

Early task-based EEG studies mostly applied event-related potentials (ERP) to evaluate the consciousness rehabilitation of DoC patients. ERP shows the potential changes induced in specific brain regions when the body receives stimuli associated with specific awareness or cognitive events. In 1990 O’Mahony et al. used EEG to evaluate comatose patients and found that auditory-related P300 potentials were associated with consciousness recovery (O’Mahony et al., 1990). Kempny et al. used DoC patients’ names, others’ names and reversed other names as ERP stimuli and detected statistically significant differences in patients’ ERP latencies to their own names versus other people’s names (Kempny et al., 2018). Wu et al. applied quantitative electroencephalography (QEEG) to monitor DoC patients’ responses to music, their own names and noise. The study showed that brain activation of patients was highest when stimulated by their own names, and QEEG index in specific stimulation states may be an indicator of consciousness rehabilitation in DoC patients (Wu et al., 2018). Li et al. (2018) found that patients’ hobby stimuli evoked stronger changes in EEG characteristics compared to music stimuli, and the changes were more pronounced in MCS patients than UWS patients. Sinitsyn et al. compared the perturbational complexity index (PCI) of DoC patients receiving TMS-EEG stimulation and found that high PCI value was related to well consciousness rehabilitation of DoC patients (Sinitsyn et al., 2020).

### 2.2. Resting-state EEG paradigm in DoC studies

Resting-state EEG detects the electrophysiological signals of the brain in DoC patients without any stimulation and acquires information about the spontaneous physiological activity of the brain. During the acquisition EEG in resting-state, the patient is usually asked to close his eyes and remain awake and relaxed (Schorr et al., 2016). Lehembre et al. (2012) found that EEG power spectrum of UWS patients increased in the delta band but decreased in the alpha band. And the connectivity (including features such as coherence and phase lag index) in theta and alpha bands was significantly lower in UWS patients than that in MCS patients. Schorr et al. further investigated the differences in EEG coherence among different brain regions of DoC patients, which provides new theoretical support for EEG coherence as a potential marker of consciousness rehabilitation (Schorr et al., 2016). Dynamic functional connectivity (DFC) is commonly used in previous fMRI analysis, Naro et al. introduced DFC into the resting-state EEG study, and found that EEG-based DFC was significantly correlated with behavioral scale scores (Naro et al., 2018). Stefan et al. applied multiple resting-state EEG analysis methods (entropy, PSD, coherence, complex network analysis, etc.) on the same dataset and combined multiple features by using a generalized linear model to classify UWS and MCS patients. The results showed that combining these features seemed to afford high prediction power and the value of AUC reached 92 ± 4% (Stefan et al., 2018).

EEG records electrophysiological signals from the human scalp and has a higher temporal resolution compared to other metabolism-based techniques (fMRI, PET, fNIRS) (Burle et al., 2015). However, EEG has a lower spatial resolution than other methods because it is susceptible to volume conduction effects (Dong et al., 2014). Compared to fMRI, EEG allows minor movements and does not require special handling of patients, thus acquisition of EEG becomes more convenient (Jain and Ramakrishnan, 2020). EEG technology was applied earlier and therefore EEG-related analytical methods are well-established. It has been demonstrated that EEG correlates with the consciousness rehabilitation of DoC patients, and task-based EEG signals exhibit better performance in characterizing the state of consciousness and cognitive abilities of the brain (Zhang et al., 2022). Part of the studies (Stefan et al., 2018; Di Gregorio et al., 2022) have turned to fuse multiple EEG features to improve the evaluating ability of DoC patients, which requires a larger number of samples. TMS-EEG provides a new means for the diagnosis and assessment of DoC patients, and the study of TMS-EEG mapping would be a potential direction for the localization of awareness in DoC patients (Wang et al., 2022; Liu et al., 2023b).

## 3. fMRI used in DoC studies

**Figure 4:**
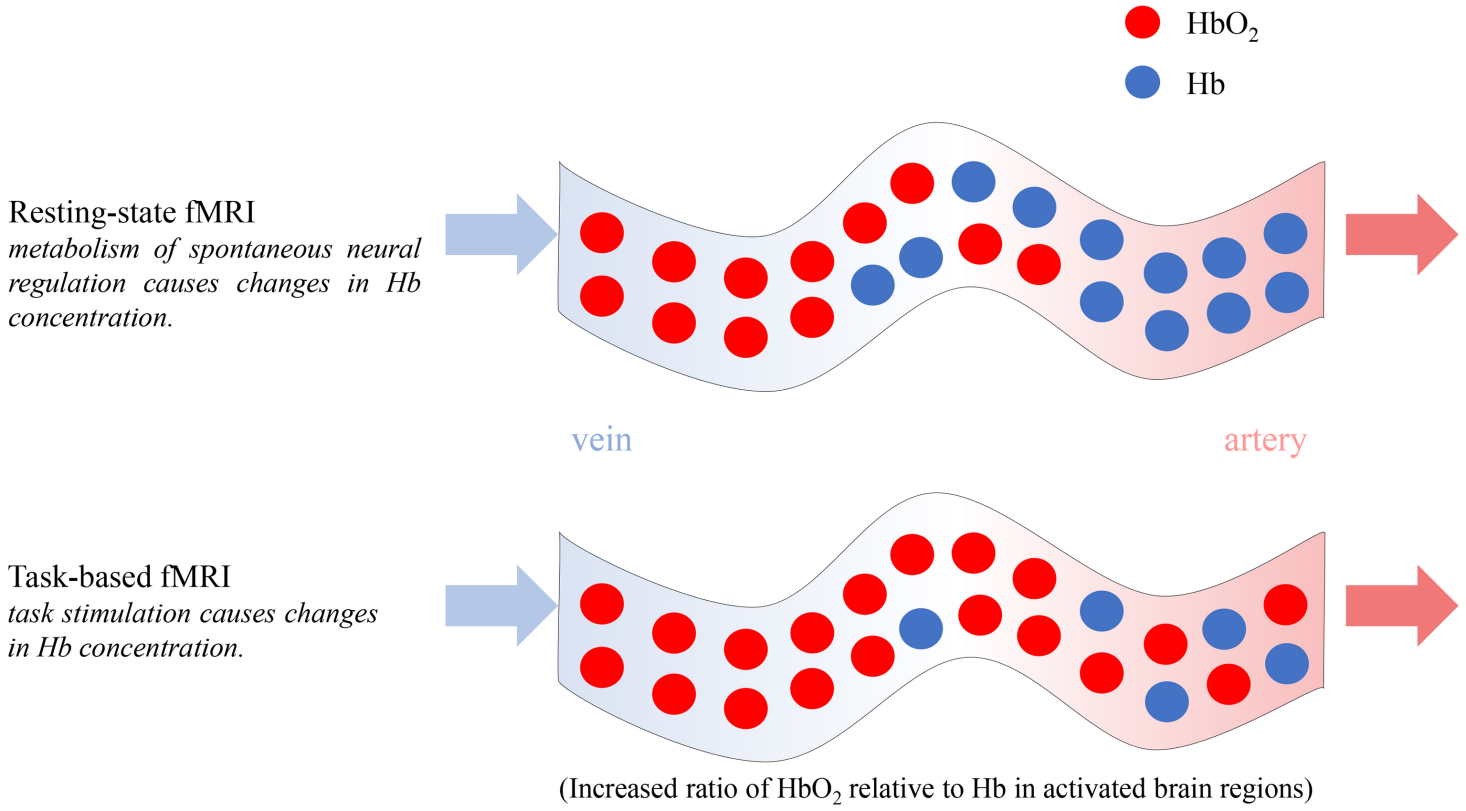
Principle of resting-state and task-based fMRI.

The development of fMRI can be traced back to 1990 and it reflects the internal brain function based on the neurovascular coupling mechanism by detecting the blood oxygenation level dependent (BOLD) signal (Figure 4). Currently, it is primarily employed in investigating cognition and mental disorders (Power et al., 2011).

### 3.1. Task-based fMRI paradigms in DoC studies

Task-based fMRI studies using auditory stimuli have been widely applied in DoC patients, as they are easier to achieve. Di et al. (2007) analyzed the brain signals using fMRI when DoC patients hearing their own name called by a familiar voice (SON-FV), and the study found a significant activation in their brains. Further studies have demonstrated that UWS/*VS* and MCS patients are capable of retaining brain responses to language and auditory stimuli, and fMRI is able to identify these responses (Fernandez-Espejo et al., 2008). Okumura et al. (2014) evaluated UWS/*VS* patients after music stimulation using fMRI and found that activation in bilateral temporal lobes was related to the recovery of consciousness. Wang et al. (2015) found that the types and volumes of auditory cortex activation induced by SON-FV were significantly correlated with the prognosis of UWS/*VS* patients, which suggested that repetitive use of the simple fMRI task may provide more reliable prognostic information. In a recent study, Boltzmann et al. (2021) scanned UWS/*VS* patients with scanner noise, preferred music and aversive auditory stimulation, and found that UWS/*VS* patients showed more significant responses to preferred music and aversive auditory stimulation, finally, the study indicated that UWS/*VS* patients require strong stimuli to elicit cerebral responses. Researches based on active fMRI paradigms have also made some progress. Liang et al. (2014) considered four imagery tasks (imagine navigating home, imagine playing tennis, imagine familiar faces, mental counting and rest) and found that different tasks activated the brain differently. Wang et al. found that the simple active fMRI “hand-raising” task can elicit brain activation in patients with DoC. Activity of the motor-related network may be taken as an indicator of high-level cognition that cannot be discerned through conventional behavioral assessment (Wang et al., 2019). Clinical implementation of fMRI motor imagery paradigms for detection of consciousness will require further development, validation, and optimization of standardized approaches to fMRI data acquisition, analysis and interpretation (Bodien et al., 2017).

### 3.2. Resting-state fMRI paradigm in DoC studies

Resting-state fMRI is a popular and advanced technique for studying the functional structure and static networks of the brain, because interaction with DoC patients and difficult experimental set-ups are not required (Jain and Ramakrishnan, 2020). Luppi et al. (2019) found that human consciousness relies on the spatiotemporal interactions between brain integration and functional diversity by comparing resting-state fMRI data of awake volunteers, propofol-anesthetized volunteers and DoC patients. Early studies have shown that the resting-state functional connectivity within the default mode network is decreased and proportional to the degree of consciousness impairment, from locked-in syndrome to MCS, *VS*, and coma patients (Vanhaudenhuyse et al., 2010). Huang et al. (2020) further analyzed resting-state fMRI signals of DoC patients and identified that human consciousness primarily depends on the default mode and the dorsal attention networks. Qin et al. (2021) analyzed fMRI data of patients with varying degrees of consciousness impairment and demonstrated that the supplementary motor area, bilateral supramarginal gyrus (part of inferior parietal lobule), supragenual/dorsal anterior cingulate cortex, and left middle temporal gyrus play important roles in maintaining brain consciousness.

fMRI is a non-invasive neuroimaging method with a high spatial resolution for accurate functional localization so it can effectively detect covert consciousness that cannot be demonstrated through clinical behavior. However, fMRI is susceptible to motion artifacts, thus has the limitations of low temporal resolution and high costs. Additionally, fMRI is not suitable for DoC patients in the Intensive Care Unit (ICU). Currently, fMRI has entered the era of “big data” with the establishment of large-scale brain datasets and the accumulation of research findings (Xia and He, 2017). The quality of fMRI data plays a critical role in the accuracy and reproducibility of research. Li et al. proposed a new denoising method called linked independent component analysis, which helps improve the accuracy and reproducibility of fMRI studies (Li et al., 2020). In addition, traditional field strengths (1.5 T and 3 T) have reached their limits in terms of spatiotemporal resolution and signal-to-noise ratio balance. However, Ultrahigh magnetic field strengths (7 T and above) allow functional imaging with even higher functional contrast-to-noise ratios for improved spatial resolution and specificity compared to traditional field strengths (1.5 T and 3 T), Which offers improved sensitivity and functional specificity for fMRI applications (Vu et al., 2017). In the future, fMRI will focus on personalized applications, standard acquisition of data and fusion of different techniques.

## 4. PET used in DoC studies

PET, as a more established technique for studying consciousness, use different markers to measure glucose metabolism, oxygen consumption, focal cerebral blood flow and the distribution of specific neurotransmitters (Hirschberg and Giacino, 2011). These markers can be used to assess the degree of residual brain function in patients with impaired consciousness (Jox et al., 2012).

PET includes two main imaging methods: cerebral perfusion imaging and cerebral metabolic imaging. Cerebral perfusion imaging employs imaging agents that are capable of traversing the blood–brain barrier and gaining entry into cerebral tissue where they exhibit stability and concentrate. Subsequently, cerebral perfusion images can be acquired by nuclear medicine imaging equipment. Patients with disorders of consciousness are severely ischemic in localized brain regions, and therefore cerebral perfusion imaging shows localized hypoperfusion or even no perfusion, which is manifested by low concentration or without concentration of the imaging agent.

### 4.1. Task-based PET paradigms in DoC studies

Task-based DoC studies mostly use H_2_^15^O-based cerebral perfusion imaging methods which assist experts to further predict the possibility of recovery in DoC patients by evaluating the specific and directional responses produced by DoC patients under external stimuli. Under the stimuli of auditory, visual and nociceptive, a dissociation occurs between the low-level activation of the primary sensory cortex and the activation of higher cortical networks in DoC patients (de Jong et al., 1997; Menon et al., 1998; Laureys et al., 2002; Bruno et al., 2010). Although task-based PET studies have yielded some results, there is still a demand for more extensive clinically responsive neuroimaging studies due to small number of studies and single cases, which make the task-based PET become a reliable tool for clinical assessment and treatment decisions in DoC patients.

### 4.2. Resting-state PET paradigm in DoC studies

In the studies of resting-state PET, brain metabolic imaging techniques are more widely used. Part of the study based on γ-aminobutyric acid receptor imaging agent observed the retention of injured brain neurons, while ligand ^11^C-labeled flumazenil (^11^C-FMZ) was used to assess neuronal integrity and activity (Schiff et al., 2002). Rudolf et al. used ^11^C-FMZ to observe the local cerebral glucose metabolism of 9 *VS* patients caused by hypoxia and found that ^11^C-FMZ PET is important in the prognostic assessment of VS. More scholars have studied resting state DoC using ^18^F-labeled FDG-PET/CT (Rudolf et al., 2002). As the main imaging agent for current PET/CT imaging, ^18^FDG can accurately reflect the glucose metabolism of organs or tissues *in vivo*. Zhao Jing et al. analyzed glucose metabolism in various brain regions of 40 *VS* patients, 12 MCS patients and 11 DoC patients with regained consciousness, and the results showed that standard uptake values in all brain regions except the brainstem were significantly different among the three groups of patients (Zhao et al., 2018). Usami et al. further found that functional preservation in the left occipital region in patients with chronic DoC might reflect an awareness of external environments, whereas extensive functional preservation in the right cerebral hemisphere might reflect communication motivation (Usami et al., 2022). Yamaki et al. investigated whether regional brain glucose metabolism assessed by ^18^F-FDG-PET in resting-state could predict voluntary movement in patients with DoC following severe traumatic brain injury. The research showed that glucose uptake in the left proximal and right proximal limb of the primary motor cortex may reflect contralateral voluntary movements (Yamaki et al., 2023); Another study found a significant difference in glucose metabolism in the thalamus, brain stem, and cerebellum between comatose and noncomatose patients acutely after TBI. The metabolic rate of glucose in these regions significantly correlated with the level of consciousness at the time of PET (Hattori et al., 2003). All these studies demonstrated the correlation between the rate of cerebral glucose metabolism and the level of consciousness.

Being sensitive to damaged brain cells, PET can accurately and objectively reflect the functional state of brain cells to assess the degree of brain cell damage. However, PET has a certain degree of radioactive damage, although the application of short-half-life radioisotopes could mitigate the adverse effects, it increases the imaging time and has a low spatial resolution, all these reasons greatly limit the application of PET/CT. In the future, taking into account both safety and accuracy, more suitable tracers will be explored to improve the effectiveness of PET in consciousness evaluation of DoC patients. Fusion with other methods is another potential direction for PET studies. PET is less sensitive to rapid and transient changes (Hermann et al., 2021), whereas EEG records brain activity in the millisecond range. Therefore, the combination of both methods may obtain greater performance in the evaluation of DoC patients.

## 5. fNIRS used in DoC studies

fNIRS is a non-invasive optical technique based on the different absorption spectral properties of oxyhemoglobin (HbO2) and deoxyhemoglobin (HbR) in human tissues. By calculating the concentration variation of both proteins, fNIRS can be used to evaluate the recovery from DoC and thus reflect the functional state of the brain (Ekkekakis, 2009).

### 5.1. Task-based fNIRS paradigms in DoC studies

Due to the portability, fNIRS devices were widely used for task-based studies (Pinti et al., 2020). fNIRS identifies patients with potential consciousness by detecting brain activity during command-driven tasks and assesses their residual awareness. Existing studies mainly use active paradigms (including motor imagery and mental arithmetic tasks). Kempny et al. evaluated the brain function of DoC patients by recording their fNIRS signals under motor imagery tasks (Kempny et al., 2016). Research has shown that MCS patients prefer to experience “typical” fNIRS responses, and their hemodynamic responses were similar to those of the control group. It confirmed the feasibility of using fNIRS to evaluate the brain function of pDoC patients through motor imagery tasks for the first time. Li et al. (2021) used fNIRS combined with an active motor imagery paradigm and demonstrated the feasibility of the paradigm to study the functional brain activity of MCS patients. The study provided new method to objectively evaluate the consciousness and residual awareness in MCS patients. The mental arithmetic task, another fNIRS research paradigm which requires higher degree of active consciousness in subjects, has been conducted (Kurz et al., 2018), however, the related studies is limited and the probability yielding positive results is relatively low, thus requiring an expanded sample study for further validation.

The fNIRS studies applying with passive paradigm have also achieved notable outcomes. Current researches focus on spinal cord stimulation (SCS). Si et al. (2018) found that when high frequencies of 70 and 100 Hz were selected for SCS, patients showed significantly enhanced cerebral hemodynamic responses in the prefrontal region and stronger functional connectivity between the prefrontal and occipital lobes. Zhang et al. found a significant increase in cerebral blood volume in the prefrontal cortex at an interstimulus interval of 30 s for SCS (Zhang et al., 2018). Part of the studies used non-traumatic stimuli. Bicciato et al. first used a frequency domain approach to investigate the variations in the oscillatory signal of fNIRS in the low frequency (LFO) and very low frequency oscillation (VLFO) spectral regions in subjects under musical stimulation (Bicciato et al., 2022). The study suggests that the use of fNIRS to identify typical patterns of brain responses to specific environmental stimulation may be a potential way to detect covert awareness in clinically unresponsive patients.

### 5.2. Resting-state fNIRS paradigm in DoC studies

There is still a relatively small amount of research based on resting-state fNIRS. Liu et al. (2023a) acquired resting-state fNIRS from 23 DoC patients and found that MCS and UWS have different patterns of topological architecture and short- and long-distance connectivity in prefrontal cortex, which confirmed that fNIRS is remarkable in distinguishing MCS and UWS. Shu et al. (2023) studied that resting-state fNIRS based functional connectivity analysis of brain networks can quantify brain communication efficiency, which would be a potential functional indicator for DoC rehabilitation.

Compared with fMRI and PET, fNIRS has the advantages of higher temporal resolution, lower cost, and high portability, as well as being less susceptible to head movements and metal implants inside the body, thus facilitating follow-up measurements and clinical application. Compared with EEG, fNIRS possesses relatively high spatial resolution, resistance to motion interference and resistance to electromagnetic interference. Since fNIRS only detects in the cerebral cortex, it has weaker sensitivity toward the deep regions of the brain and therefore cannot detect information in the deeper part of the brain; moreover, the temporal and spatial characteristics make its signal processing and data analysis algorithms lack uniform standards.

**Figure 5:**
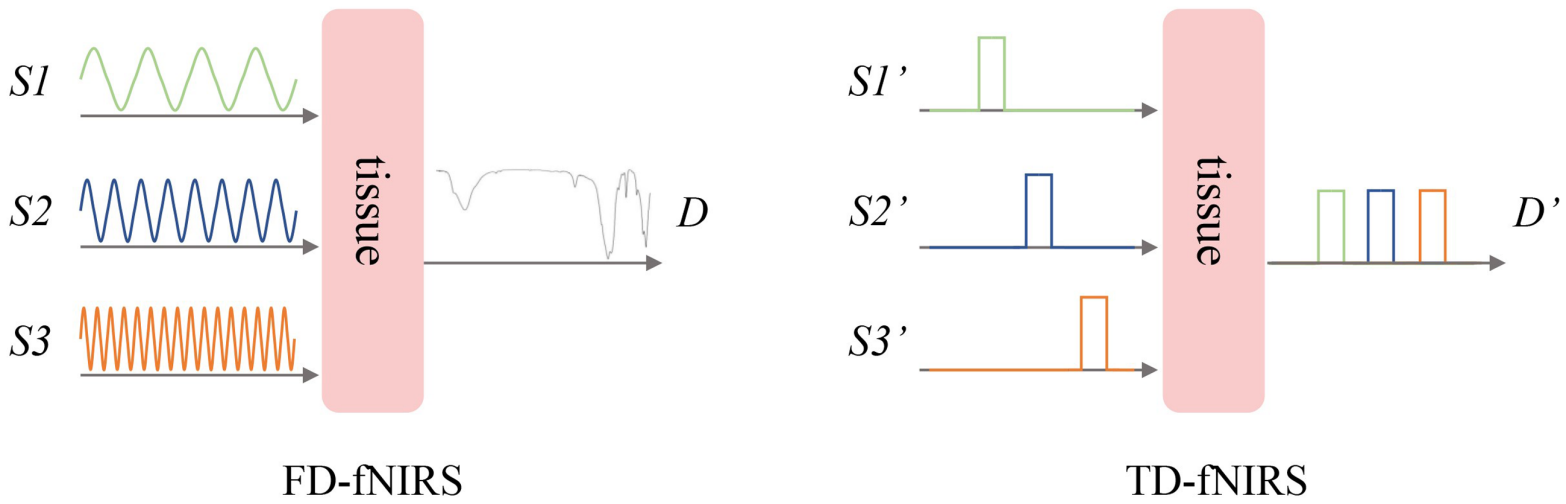
Frequency-domain and time-domain fNIRS.

In the future, fNIRS is expected to be fused with other neuroimaging methods to achieve multimodal neuro-detection for more accurate and comprehensive evaluation. In addition, the use of time-domain-based functional near-infrared spectroscopy (TD-fNIRS) and frequency-domain-based functional near-infrared spectroscopy (FD-fNIRS) can improve the sensitivity of fNIRS toward the deep brain (Figure 5). More sophisticated machine learning approaches such as artificial neural networks could also help improve the sensitivity of fNIRS (Abdalmalak et al., 2021). Finally, researchers should establish a unified and effective method for fNIRS data analysis for the promotion and application of fNIRS.

## 6. Multi-modal neuroimaging methods used in DoC studies

Different neuroimaging methods have distinct characteristics, yet no single method combines all the advantages (Table 2). Neuroimaging methods using a single modality can no longer meet the requirements of researchers and multi-modal methods have been gradually applied in recent research. Multi-modal methods can achieve complementary advantages of different methods, thereby uncovering significant relationships that cannot be detected by employing a single modality alone.

**Table 2:** Characteristics of different neuroimaging methods.

Neuroimaging methods	Spatial resolution	Temporal resolution	Cost	Accessibility	Risk
EEG	Low	High	Affordable	High	Non-invasive
fMRI	High	Low	High	Low	Non-invasive
PET	Medium	Low	High	Low	Radioactive damage
fNIRS	Medium	Medium	Low	High	Non-invasive

**Figure 6:**
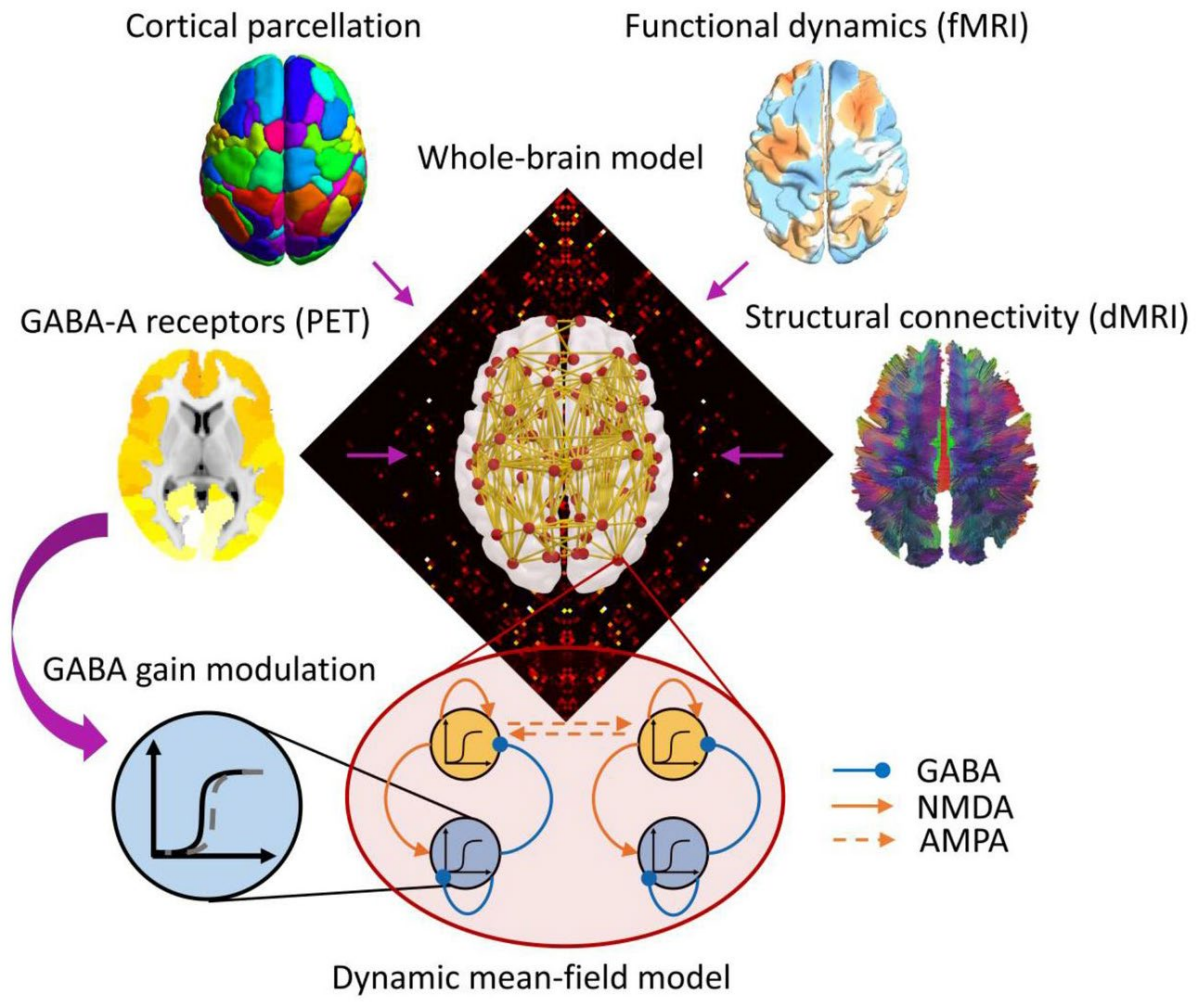
Multimodal study based on PET and MRI.

PET/MRI can evaluate brain structure and metabolism at the same time, helping doctors to increase the probability of detecting (spared) functional connectivity (Cavaliere et al., 2018). Luppi et al. (2022) leveraged a neurobiologically realistic dynamic mean field model informed by multimodal neuroimaging including fMRI, diffusion MRI and PET. The study provides a new idea for the evaluation of DoC recovery using PET/MRI (Figure 6). Hermann et al. (2021) discovered that the combination of FDG-PET and EEG accurately identified almost all MCS patients and patients with 6-month recovery of command-following, outperforming each technique taken in isolation. The fusion application of fMRI and EEG can precisely locate brain activity in time and space due to the complementary nature of their spatiotemporal resolution (Chatzichristos et al., 2022). Based on combined EEG and fMRI features, Amiri et al. (2023) found that models which use machine-learning algorithms (SVM and Random Forest) predicted consciousness levels with improved positive predictive value and sensitivity. Despite the high spatial resolution of fMRI, the bulky size of fMRI equipment makes it difficult to collect data in ICU. While maintaining portability, fNIRS can be collected simultaneously with EEG to improve its sensitivity, which makes multi-modal detection more convenient. EEG measures an electrical process which does not interfere with the light signals measured by fNIRS. The improved spatial resolution offered by fNIRS can provide information regarding the active source location, thus complementing EEG findings (Rupawala et al., 2018). Othman et al. (2021) found that neurovascular coupling between NIRS oxyhemoglobin (0.07–0.13 Hz) and EEG band-power (1–12 Hz) signals at frontal areas was sensitive and prognostic to changing consciousness levels. The study suggests that NIRS–EEG may be worth exploring as an add-on to multimodal neuromonitoring and recovery of neurovascular coupling after acute brain injury may herald recovery of consciousness.

The human brain entertains rich spatiotemporal dynamics, and adopting multimodal methods allows for the integration of both temporal and spatial characteristics of brain signals, thus facilitating a more comprehensive understanding of the brain. However, the application of multimodal neuroimaging methods requires substantial data, which increases the workload of researchers. The development of more integrated and portable acquisition devices holds the potential to ameliorate the situation. Furthermore, Recent research based on multimodal neuroimaging methods primarily focuses on the resting-state paradigm, future studies could consider incorporating task-based paradigms to detect the temporal coupling of different neural signals.

## 7. Conclusion

Although there is still wide disagreement about what exactly consciousness is, neuroimaging methods have been proven to be effective in consciousness rehabilitation evaluation. Especially in patients without command following or other signs of consciousness at the bedside, neuroimaging methods are useful to detect covert consciousness (if present), avoid misdiagnosis and help patients to communicate with the outside world. PET can accurately and objectively reflect the functional status of brain cells. However, compared with other methods, it has a certain degree of radioactive damage, which limits the development of related studies. Considering both safety and accuracy, exploring tracers with greater performance is a potential research direction. Among lots of neuroimaging methods, EEG and fMRI are more widely used. EEG has made great progress in the evaluation and classification of DoC patients in task-based paradigms due to its high temporal resolution, whereas fMRI has a unique advantage in functional brain networks due to its high spatial resolution. EEG has a lower spatial resolution than other methods because it is susceptible to volume conduction effects. High-density EEG paradigms appear to have a high specificity but very low sensitivity for the detection of covert consciousness. fNIRS has a moderate spatial and temporal resolution, and has become a burgeoning tool for the evaluation of DoC because it is more portable and economical. Multi-modal methods can achieve complementary advantages of different methods. However, the challenges associated with data acquisition has hindered its development to some extent. As neuroimaging devices become wireless, integrated and portable, multi-modal neuroimaging method is expected to have a broader application in the evaluation of consciousness rehabilitation and will drive new advancements in brain science research.

## Author contributions

JW, XG, and ZX: writing – original draft preparation and visualization. FS and YY: writing – review and editing, supervision, project administration, and funding acquisition. All authors have read and agreed to the published version of the manuscript.

## Funding

This research was funded by the Key R&D Program of Zhejiang, grant number 2023C03081.

## Conflict of interest

The authors declare that the research was conducted in the absence of any commercial or financial relationships that could be construed as a potential conflict of interest.

## Publisher’s note

All claims expressed in this article are solely those of the authors and do not necessarily represent those of their affiliated organizations, or those of the publisher, the editors and the reviewers. Any product that may be evaluated in this article, or claim that may be made by its manufacturer, is not guaranteed or endorsed by the publisher.
